# Methylation of *SSTR4* promoter region in multiple mental health disorders

**DOI:** 10.3389/fgene.2024.1431769

**Published:** 2024-07-11

**Authors:** Rongrong Zhao, Huihui Shi, Yanqiu Wang, Shuaiyu Zheng, Yahui Xu

**Affiliations:** ^1^ The First Affiliated Hospital and College of Clinical Medicine of Henan University of Science and Technology, Luoyang, China; ^2^ The Second Affiliated Hospital of Xinxiang Medical University, Xinxiang, China

**Keywords:** *SSTR4*, schizophrenia, major depressive disorder, bipolar disorder, alcohol use disorder, DNA methylation, epigenetic clock

## Abstract

The existence of a shared genetic basis for mental disorders has long been documented, yet research on whether acquired epigenetic modifications exhibit common alterations across diseases is limited. Previous studies have found that abnormal methylation of cg14631053 at the *SSTR4* promoter region mediates the onset of alcohol use disorder. However, whether aberrant methylation of the *SSTR4* gene promoter is involved in other mental health disorders remains unclear. In this study, leveraging publicly available data, we identified that changes in methylation of cg14631053 from the *SSTR4* promoter region are involved in the development of bipolar disorder and schizophrenia. Furthermore, the direction of methylation changes in the *SSTR4* promoter region is disease-specific: hypomethylation is associated with the onset of bipolar disorder and schizophrenia, rather than major depressive disorder. Methylation levels of cg14631053 correlate with chronological age, a correlation that can be disrupted in patients with mental health disorders including schizophrenia and bipolar disorder. In conclusion, *SSTR4* promoter methylation may serve as a marker for identifying bipolar disorder and schizophrenia, providing insights into a transdiagnostic mechanism for precision medicine in the future.

## Introduction

Epigenetic modifications are increasingly recognized as pivotal factors in the pathogenesis of severe mental disorders such as schizophrenia, major depressive disorder, and bipolar disorder (Nohesara et al., 2023). Shared epigenetic alterations may underlie the co-occurrence of these disorders, shedding light on the intricate interplay between genetic predisposition and environmental influences (Grezenko et al., 2023). Recent investigations have revealed DNA methylation aberrations affecting multiple genes across various mental disorders, implying common underlying mechanisms (Abdolmaleky et al., 2021). Notably, DNA hypermethylation of RELN has been consistently observed in postmortem brain samples of patients with schizophrenia, major depressive disorder, and bipolar disorder, indicating a potential shared epigenetic signature (Colită et al., 2024). These epigenetic changes have been implicated in mediating the impact of environmental risk factors associated with the development of mental disorders, including infections, malnutrition, prenatal stress, and childhood adversities (Colită et al., 2024).

The somatostatin receptor subtype 4 (*SSTR4*) gene has emerged as a significant focus in the investigation of mental health disorders, including schizophrenia, depression, and bipolar disorder, with research implicating its involvement in their pathogenesis (Berent et al., 2017; Zhang et al., 2020; Adamcyzk et al., 2021). Variations in *SSTR4* expression or function are suggested to contribute to symptom development, as evidenced by findings linking a functional polymorphism (rs2567608) to increased suicide risk in individuals with alcohol dependence (Berent et al., 2017). Aging-related expression of *SSTR4* has been reported in patients with Alzheimer’s disease (Grosser et al., 2014). Furthermore, reduced *SSTR4* expression in brain regions like the hippocampus and amygdala in schizophrenia patients and animal models may disrupt neural circuitry associated with aversion and reward processing (Zhang et al., 2020). Moreover, preclinical studies indicate the potential of *SSTR4* agonists as treatments for anxiety and depression, possibly through normalization of dysregulated neurocircuitry (Scheich et al., 2016; Adamcyzk et al., 2021).

Epigenetic aging, gauged through DNA methylation patterns, has emerged as a robust indicator of biological age, distinct from chronological age, encapsulated by “epigenetic clocks.” This concept offers a novel perspective on aging and its association with health outcomes, including mental health disorders, with recent research delving into the link between accelerated epigenetic aging and conditions such as depression, anxiety, and schizophrenia (Klopack et al., 2022; Protsenko et al., 2023; Yusupov et al., 2023; Colită et al., 2024). The relevance of epigenetic aging to mental health lies in its potential to reflect cumulative life stress and biological responses to environmental and psychosocial factors, with adverse experiences accelerating epigenetic aging and heightening susceptibility to psychiatric conditions (Zannas, 2019; Klopack et al., 2022; Yusupov et al., 2023). Mechanistically, these associations are underpinned by inflammatory pathways, cellular aging processes, and gene expression alterations crucial in stress regulation and psychiatric pathophysiology (Zannas, 2019; Klopack et al., 2022; Yusupov et al., 2023). Such findings underscore epigenetic aging’s utility as a biomarker for identifying at-risk individuals, potentially facilitating early intervention and tailored therapies while offering insights into novel treatment and prevention avenues, thereby unraveling the intricate connections between genetics, environment, and mental health (Klopack et al., 2022; Protsenko et al., 2023; Yusupov et al., 2023; Colită et al., 2024).

Our previous study suggested abnormal methylation of *SSTR4* in promoter region (cg14631053) as a risk for alcohol use disorder development (Zhao et al., 2022). However, whether this abnormality is associated with other mental health disorders remains unclear and is worth to explore that further expanding our understanding of mechanism and informed clinical development for treatment. In this study, we employed individuals with schizophrenia, major depressive disorder, bipolar disorder to instigate whether the abnormal methylation of cg14631053 can be observed in these mental health disorders.

## Method and materials

### Participants

In this study, our investigation relied on publicly available datasets from GEO databases. Specifically, we analyzed the dataset GSE112179 (frontal cortex) to examine individuals diagnosed with schizophrenia (*n* = 35) alongside healthy controls (*n* = 33) (Pai et al., 2019). For individuals with bipolar disorder (*n* = 32) and corresponding healthy controls (*n* = 32), we utilized the dataset GSE129428 collected from hippocampus (Fries et al., 2020). Similarly, for individuals diagnosed with major depressive disorder (*n* = 37) and their respective healthy controls (*n* = 38), we employed the dataset GSE88890 collected from Brodmann Area 11 and Brodmann Area 25 (Murphy et al., 2017). All datasets are collected in post-mortem brains.

### Estimation and adjustment of confounders

We used the DNA Methylation Age Calculator to estimate the cell proportion from each dataset. We included sex, age, cell proportion, and death time in the linear model by R package limma (Ritchie et al, 2015) to reduce the potential confounding.

### Chronological aging analysis

Chronological aging analysis was conducted employing two approaches: Firstly, in healthy controls (n_brain_ = 194, n_blood_ = 1,615), *SSTR4* was assessed utilizing the EWAS Open Platform, encompassing the EWAS Atlas, EWAS Data Hub, and EWAS Toolkit, to investigate the relationship between chronological age and methylation level of cg14631053 through Pearson’s correlation. Secondly, in individuals with mental health disorders, Pearson correlation was performed between chronological age and methylation level of cg14631053 within each dataset for each respective disease.

### Methylation quantitative trait loci analysis

We conducted an analysis of methylation quantitative trait loci (meQTLs) for CpG sites originating from *SSTR4* using the MeQTL EPIC Database (https://epicmeqtl.kcl.ac.uk/) (Villicaña et al., 2023) and mQTLdb (http://www.mqtldb.org/) (Gaunt et al., 2016). The genetic variant that acts as meQTL will be further clumped due to the linkage disequilibrium (LD, *R* ^2^ or D` > 0.2).

### LD trait analysis

To confirm what trait the meQTL may involve, LD trait analysis was conducted on LDlink Platform (https://ldlink.nih.gov/). We use the clumped meQTLs to examine the genetic variant affected trait from the summary of genome-wide association study.

## Statistics

All statistical analyses are conducted in software R (version 4.1.1). Student’s *t*-test was used to compare the difference in mean between two groups. Pearson correlation was to used to determine the correlation between two numerical variables. A threshold of *p* < 0.05 was considered as the significant difference.

## Results

### CpG site cg14631053 hypomethylated in bipolar disorder and schizophrenia, but not major depressive disorder

**FIGURE 1:**
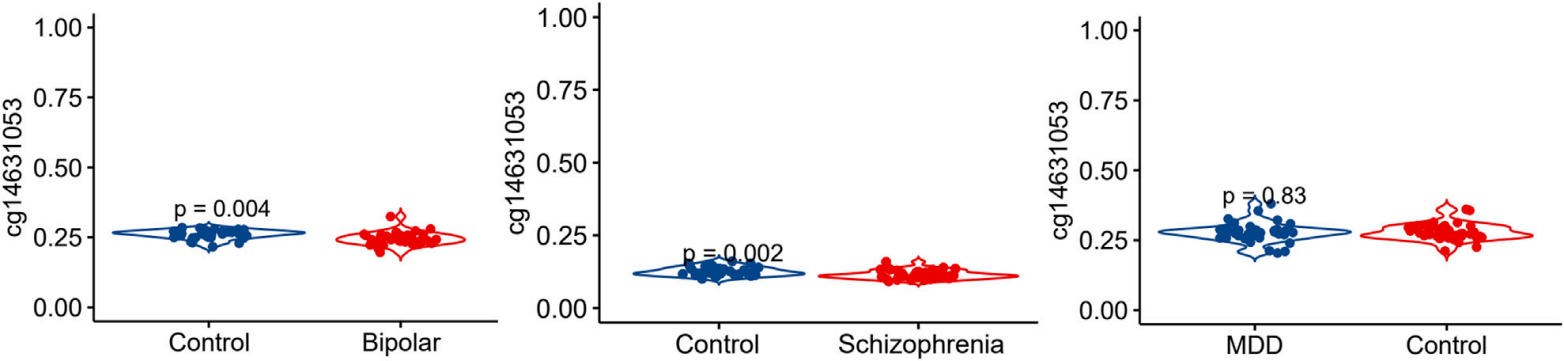
Methylation level of cg14631053 comparison among participants with mental health disorders and healthy control. Violin plot for comparison among participants with mental health disorders and healthy control (Student’s *t*-test, two tailed). Significant differences (*p* < 0.05) in mean compared heathy control were observed in patients with bipolar disorder (left panel) and schizophrenia (middle panel).

We conducted an analysis of the methylation status of cg01471923 within the *SSTR4* promoter region across individuals with mental health disorders and a healthy control group. Our findings reveal a notable decrease in methylation levels of cg01471923 among patients diagnosed with bipolar disorder (Figure 1A, *p* = 0.004) or schizophrenia (Figure 1B, *p* = 0.002), whereas no significant variation was observed in individuals with major depressive disorder (Figure 1C). Additionally, we explored other CpG sites within the *SSTR4* gene, finding no statistically significant distinctions between cases (bipolar disorder, schizophrenia, or MDD) and healthy controls, with the exception of cg18197392 in bipolar disorder (*p* = 0.042, Supplementary Figures S2–S4). In addition, rs13045080 served as a *cis* methylation quantitative trait locus (QTL) for cg18197392 according to the MeQTL EPIC Database (*p* = 2.03 × 10^−8^). GTEx database confirmed rs13045080 showed linkage disequilibrium the expression QTL for *SSTR4* gene (see Supplementary Figure S5; Supplementary Table S1).

### Chronological age is associated with methylation level of cg14631053

**FIGURE 2:**
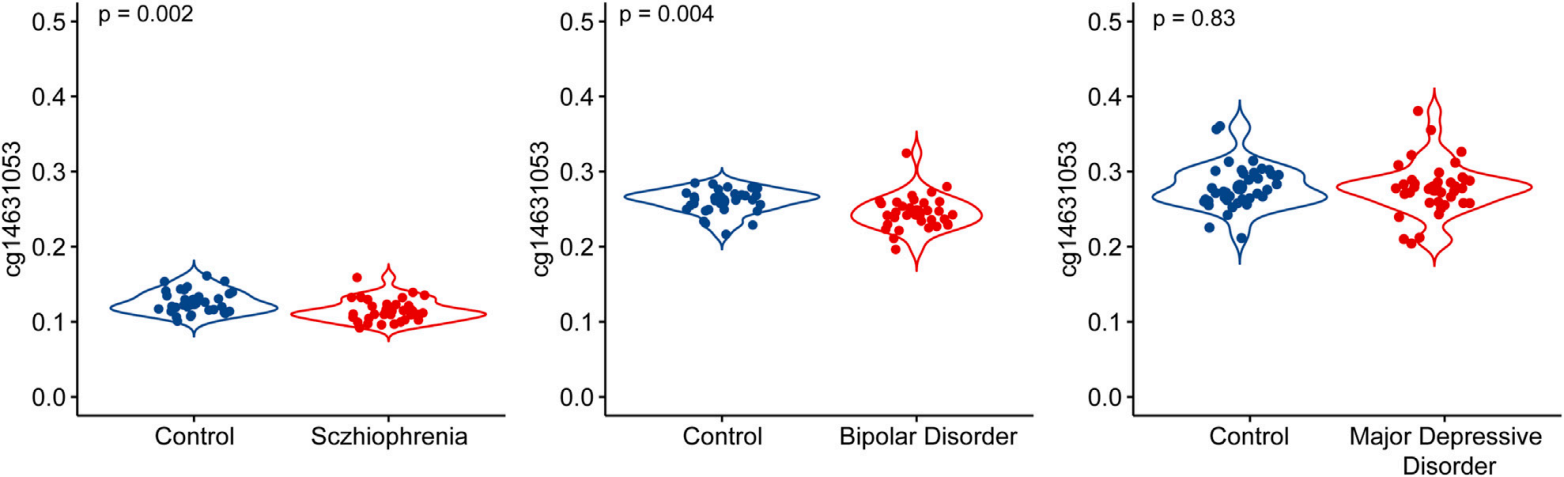
Correlation between chronological age and methylation level of cg14631053 in healthy control in blood tissue and brain tissue.

We investigated the association between chronological age and the methylation level of cg14631053 in both whole blood and the prefrontal cortex of humans. Our analysis revealed a significant and positive correlation between the methylation level of cg14631053 and chronological age in both tissues from healthy control (Figure 2, whole blood: *r* ^2^_female_ = 0.22, *r* ^2^_male_ = 0.27; prefrontal cortex: *r* ^2^_female_ = 0.27, *r* ^2^_male_ = 0.21).

### Disrupted association of chronological age and methylation level of cg14631053 in mental health disorders

**FIGURE 3:**
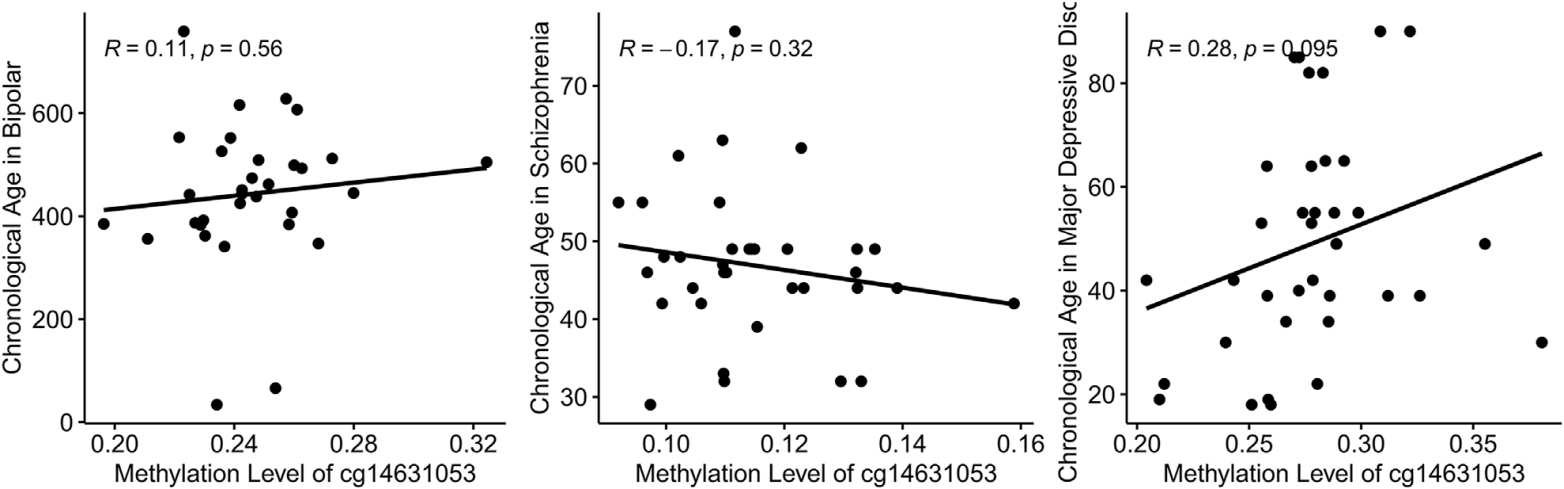
Correlation between chronological age and methylation level of cg14631053 in mental health disorders.

In this study, we initially established a correlation between chronological age and the methylation level of cg14631053 in healthy individuals. Subsequently, we sought to ascertain if this correlation persisted in individuals with mental health disorders. Utilizing Pearson’s correlation analysis, we evaluated the relationship between chronological age and cg14631053 methylation in bipolar disorder, schizophrenia, and major depressive disorder. Our findings indicate a lack of significant correlation in these psychiatric conditions (Figure 3, positive correlation in patients with bipolar disorder and patients with major depressive disorder and negative correlation in patients with schizophrenia). Furthermore, we observed a notable hypomethylation of cg14631053 in patients with schizophrenia or bipolar disorder compared to healthy controls. This aberration led to a diminished correlation coefficient between chronological age and cg14631053 methylation in these disorders, as opposed to major depressive disorder alone.

## Discussion

The study investigated the association between chronological age and the methylation level of cg14631053 in healthy individuals, finding a significant correlation in both whole blood and prefrontal cortex tissues. However, this correlation did not persist in individuals with mental health disorders, including bipolar disorder, schizophrenia, and major depressive disorder. Notably, hypomethylation of cg14631053 was observed in patients with schizophrenia or major depressive disorder compared to healthy controls, resulting in a reduced correlation coefficient between chronological age and cg14631053 methylation in these disorders. These findings suggest disrupted epigenetic regulation associated with aging in schizophrenia and major depressive disorder as well as a transdiagnostic evidence in schizophrenia, bipolar disorder, and alcohol use disorder for further investigation.

Somatostatin (SST), initially identified as a hypothalamic factor inhibiting growth hormone release, acts as a neurotransmitter or neuromodulator with primarily inhibitory action in the central nervous system (CNS) (Llona and Eugenín, 2005). Distributed throughout the CNS and periphery, somatostatin and its receptors (SSTR) are implicated in various biological functions (Llona and Eugenín, 2005). In brain development, SST influences synaptogenesis, neuroblast proliferation, and axonal pathfinding, indicating both trophic and apoptotic roles (Liguz-Lecznar et al., 2016). Somatostatin-containing neurons regulate cortex assembly, functional maturation, and contribute significantly to nervous system activity and plasticity (Liguz-Lecznar et al., 2016). Implicated in motor activity, sleep, sensory processes, cognitive functions, and neuronal plasticity, alterations in the somatostatinergic system are associated with brain disorders such as affective disorders, epilepsy, and Alzheimer’s disease (Liguz-Lecznar et al., 2016). Despite extensive research, the mechanisms underlying somatostatin’s interaction with neurotransmitters to modulate excitability and neuronal network responses remain incompletely understood, highlighting the need for further investigation (Llona and Eugenín, 2005). Abnormalities in somatostatin are evident in schizophrenia, major depressive disorder, and bipolar disorder, manifesting as decreased cerebrospinal fluid (CSF) somatostatin levels and reduced somatostatin immunoreactivity across cortical and subcortical regions in both schizophrenia and major depressive disorder (Rubinow, 1986; Lin and Sibille, 2013). In major depressive disorder, there is also a decrease in the number and density of somatostatin-expressing neurons in the hippocampus (Lin and Sibille, 2013). Similarly, bipolar disorder is characterized by reduced somatostatin cellular density in the caudal entorhinal cortex, diminished numbers of somatostatin-expressing neurons in the hippocampus, and elevated CSF somatostatin levels during manic states, along with decreased somatostatin gene expression in the dorsolateral prefrontal cortex and hippocampus (Lin and Sibille, 2013).

*SSTR4*, a subtype of somatostatin receptor, plays a crucial role in the central nervous system (CNS), exhibiting distinct expression patterns in specific CNS cells, such as hepatic oval cells (HOCs) during liver regeneration (Jung et al., 2006). In liver regeneration, *SSTR4* functions as a chemoattractant, influencing HOC migration (Jung et al., 2006). Moreover, it is highly expressed in stress-related brain regions like the hippocampus and amygdala, modulating emotional behavior and stress responses (Scheich et al., 2017). Activation of *SSTR4* exerts anxiolytic and antidepressant-like effects by enhancing stress-responsiveness in specific brain regions (Scheich et al., 2016). Additionally, *SSTR4* activation normalizes stress-induced glutamate release in the amygdala, crucial for regulating stress-related behaviors (Scheich et al., 2016). Its involvement in these processes is mediated through intracellular signaling pathways, influencing neurotransmitter release and neuronal excitability (Patel, 1999). Genetic deletion of *SSTR4* results in increased anxiety and depression-like behaviors, highlighting its critical role in emotional stability under stress (Scheich et al., 2016; 2017). Moreover, *SSTR4* activation triggers cell proliferation through STAT3 phosphorylation, indicating its significance in cellular processes (Song et al., 2021). Furthermore, *SSTR4* agonism normalizes stress-induced glutamate release in the basolateral amygdala, suggesting a regulatory role in CNS stress responses (Adamcyzk et al., 2021). However, there is no specific information regarding *SSTR4* abnormalities in schizophrenia, major depressive disorder, or bipolar disorder. Nonetheless, abnormal methylation of cg01471923, first reported in patients with alcohol use disorder (Zhao et al., 2022), has been replicated in patients with schizophrenia and bipolar disorder, expanding understanding of *SSTR4* and providing transdiagnostic evidence for targeted clinical developments.

Schizophrenia exhibits accelerated epigenetic aging, rendering the brain biologically older by several years compared to chronological age, a phenomenon observed early in the illness trajectory (Segura et al., 2023). In contrast, bipolar disorder presents a mixed picture, with studies showing varied findings, including epigenetic age acceleration, deacceleration, or no deviation from controls, with potential disparities across life stages, particularly in older adulthood (Segura et al., 2023). Major depressive disorder is consistently linked to accelerated epigenetic aging in both blood and brain tissue, with factors such as symptom severity and childhood trauma exacerbating this acceleration (Han et al., 2018). We found the correlation between methylation level of cg01471923 and chronological age was disrupted in schizophrenia, major depressive disorder, and bipolar disorder, with a different direction, suggesting a unique epigenetic aging picture of each disease may associated with their onset.

In conclusion, our study reveals disrupted epigenetic regulation associated with aging in schizophrenia and bipolar disorder. Considering our previous finding, this study offered transdiagnostic insights from abnormal methylation of *SSTR4* promoter region for schizophrenia, bipolar disorder and alcohol use disorder.

Scatter plot for Pearson correlation between chronological age and cg14631053 in healthy control in brain tissue (upper panel, *n* = 194) and blood tissue (lower panel, *n* = 1,615).

Scatter plot for Pearson correlation between chronological age and cg14631053 in each mental health disorder. No significant correlation was observed.

## Data Availability

The original contributions presented in the study are included in the article/Supplementary Material, further inquiries can be directed to the corresponding authors.

## References

[B1] Abdolmaleky H. M. Zhou J.-R. Thiagalingam S. (2021). Cataloging recent advances in epigenetic alterations in major mental disorders and autism. Epigenomics 13, 1231–1245. 10.2217/epi-2021-0074 34318684 PMC8738978

[B2] Adamcyzk I. Kúkeľová D. Just S. Giovannini R. Sigrist H. Amport R. (2021). Somatostatin receptor 4 agonism normalizes stress-related excessive amygdala glutamate release and pavlovian aversion learning and memory in rodents. Biol. Psychiatry Glob. Open Sci. 2, 470–479. 10.1016/j.bpsgos.2021.11.006 36324659 PMC9616361

[B3] Berent D. Emilien G. Podgórski M. Kusideł E. Kulczycka-Wojdala D. Szymańska B. (2017). *SSTR4*, childhood adversity, self-efficacy and suicide risk in alcoholics. Transl. Neurosci. 8, 76–86. 10.1515/tnsci-2017-0013 28924491 PMC5597949

[B4] Colită C.-I. Udristoiu I. Ancuta D.-L. Hermann D. M. Colita D. Colita E. (2024). Epigenetics of ageing and psychiatric disorders. JIN 23, 13. 10.31083/j.jin2301013 38287856

[B5] Fries G. R. Bauer I. E. Scaini G. Valvassori S. S. Walss-Bass C. Soares J. C. (2020). Accelerated hippocampal biological aging in bipolar disorder. Bipolar Disord. 22, 498–507. 10.1111/bdi.12876 31746071

[B6] Gaunt T. R. Shihab H. A. Hemani G. Min J. L. Woodward G. Lyttleton O. (2016). Systematic identification of genetic influences on methylation across the human life course. Genome Biol. 17, 61. 10.1186/s13059-016-0926-z 27036880 PMC4818469

[B7] Grezenko H. Ekhator C. Nwabugwu N. U. Ganga H. Affaf M. Abdelaziz A. M. (2023). Epigenetics in neurological and psychiatric disorders: a comprehensive review of current understanding and future perspectives. Cureus 15, e43960. 10.7759/cureus.43960 37622055 PMC10446850

[B8] Grosser C. Neumann L. Horsthemke B. Zeschnigk M. van de Nes J. (2014). Methylation analysis of *SST* and *SSTR4* promoters in the neocortex of Alzheimer’s disease patients. Neurosci. Lett. 566, 241–246. 10.1016/j.neulet.2014.02.046 24602981

[B9] Han L. K. M. Aghajani M. Clark S. L. Chan R. F. Hattab M. W. Shabalin A. A. (2018). Epigenetic aging in major depressive disorder. AJP 175, 774–782. 10.1176/appi.ajp.2018.17060595 PMC6094380 29656664

[B10] Jung Y. Oh S.-H. Zheng D. Shupe T. D. Witek R. P. Petersen B. E. (2006). A potential role of somatostatin and its receptor *SSTR4* in the migration of hepatic oval cells. Lab. Invest. 86, 477–489. 10.1038/labinvest.3700410 16534498

[B11] Klopack E. T. Crimmins E. M. Cole S. W. Seeman T. E. Carroll J. E. (2022). Accelerated epigenetic aging mediates link between adverse childhood experiences and depressive symptoms in older adults: results from the Health and Retirement Study. SSM Popul. Health 17, 101071. 10.1016/j.ssmph.2022.101071 35313610 PMC8933834

[B12] Liguz-Lecznar M. Urban-Ciecko J. Kossut M. (2016). Somatostatin and somatostatin-containing neurons in shaping neuronal activity and plasticity. Front. Neural Circuits 10, 48. 10.3389/fncir.2016.00048 27445703 PMC4927943

[B13] Lin L.-C. Sibille E. (2013). Reduced brain somatostatin in mood disorders: a common pathophysiological substrate and drug target? Front. Pharmacol. 4, 110. 10.3389/fphar.2013.00110 24058344 PMC3766825

[B14] Llona I. Eugenín J. (2005). Central actions of somatostatin in the generation and control of breathing. Biol. Res. 38, 347–352. 10.4067/S0716-97602005000400006 16579516

[B15] Murphy T. M. Crawford B. Dempster E. L. Hannon E. Burrage J. Turecki G. (2017). Methylomic profiling of cortex samples from completed suicide cases implicates a role for PSORS1C3 in major depression and suicide. Transl. Psychiatry 7, e989. 10.1038/tp.2016.249 28045465 PMC5545719

[B16] Nohesara S. Abdolmaleky H. M. Thiagalingam S. (2023). Epigenetic aberrations in major psychiatric diseases related to diet and gut microbiome alterations. Genes 14, 1506. 10.3390/genes14071506 37510410 PMC10379841

[B17] Pai S. Li P. Killinger B. Marshall L. Jia P. Liao J. (2019). Differential methylation of enhancer at IGF2 is associated with abnormal dopamine synthesis in major psychosis. Nat. Commun. 10, 2046. 10.1038/s41467-019-09786-7 31053723 PMC6499808

[B18] Patel Y. C. (1999). Somatostatin and its receptor family. Front. Neuroendocrinol. 20, 157–198. 10.1006/frne.1999.0183 10433861

[B19] Protsenko E. Wolkowitz O. M. Yaffe K. (2023). Associations of stress and stress-related psychiatric disorders with GrimAge acceleration: review and suggestions for future work. Transl. Psychiatry 13, 142–211. 10.1038/s41398-023-02360-2 37130894 PMC10154294

[B20] Rubinow D. R. (1986). Cerebrospinal fluid somatostatin and psychiatric illness. Biol. Psychiatry 21, 341–365. 10.1016/0006-3223(86)90163-0 2869790

[B21] Scheich B. Csekő K. Borbély É. Ábrahám I. Csernus V. Gaszner B. (2017). Higher susceptibility of somatostatin 4 receptor gene-deleted mice to chronic stress-induced behavioral and neuroendocrine alterations. Neuroscience 346, 320–336. 10.1016/j.neuroscience.2017.01.039 28161436

[B22] Scheich B. Gaszner B. Kormos V. László K. Ádori C. Borbély É. (2016). Somatostatin receptor subtype 4 activation is involved in anxiety and depression-like behavior in mouse models. Neuropharmacology 101, 204–215. 10.1016/j.neuropharm.2015.09.021 26387439

[B23] Segura A. G. de la Serna E. Sugranyes G. Baeza I. Valli I. Díaz-Caneja C. (2023). Epigenetic age deacceleration in youth at familial risk for schizophrenia and bipolar disorder. Transl. Psychiatry 13, 155–158. 10.1038/s41398-023-02463-w 37156786 PMC10167217

[B24] Song Y.-H. Yoon J. Lee S.-H. (2021). The role of neuropeptide somatostatin in the brain and its application in treating neurological disorders. Exp. Mol. Med. 53, 328–338. 10.1038/s12276-021-00580-4 33742131 PMC8080805

[B25] Villicaña S. Castillo-Fernandez J. Hannon E. Christiansen C. Tsai P.-C. Maddock J. (2023). Genetic impacts on DNA methylation help elucidate regulatory genomic processes. Genome Biol. 24, 176. 10.1186/s13059-023-03011-x 37525248 PMC10391992

[B26] Yusupov N. Dieckmann L. Erhart M. Sauer S. Rex-Haffner M. Kopf-Beck J. (2023). Transdiagnostic evaluation of epigenetic age acceleration and burden of psychiatric disorders. Neuropsychopharmacol 48, 1409–1417. 10.1038/s41386-023-01579-3 PMC10354057 37069357

[B27] Zannas A. S. (2019). Epigenetics as a key link between psychosocial stress and aging: concepts, evidence, mechanisms. Dialogues Clin. Neurosci. 21, 389–396. 10.31887/DCNS.2019.21.4/azannas 31949406 PMC6952744

[B28] Zhang Y. You X. Li S. Long Q. Zhu Y. Teng Z. (2020). Peripheral blood leukocyte RNA-seq identifies a set of genes related to abnormal psychomotor behavior characteristics in patients with schizophrenia. Med. Sci. Monit. 26, e922426–e922431. 10.12659/MSM.922426 32038049 PMC7032534

[B29] Zhao R. Shi H. Yin J. Sun Z. Xu Y. (2022). Promoter specific methylation of *SSTR4* is associated with alcohol dependence in han Chinese males. Front. Genet. 13, 915513. 10.3389/fgene.2022.915513 35754825 PMC9218598

