# Supplementary material for: Methylation of SSTR4 promoter region in multiple mental health disorders
Source: Front Genet. 2024 Jul 11;15:1431769. doi: 10.3389/fgene.2024.1431769 (PMC11269100; doi:10.3389/fgene.2024.1431769)
Supplement: Supplementary file 2 [file Table1.DOCX]

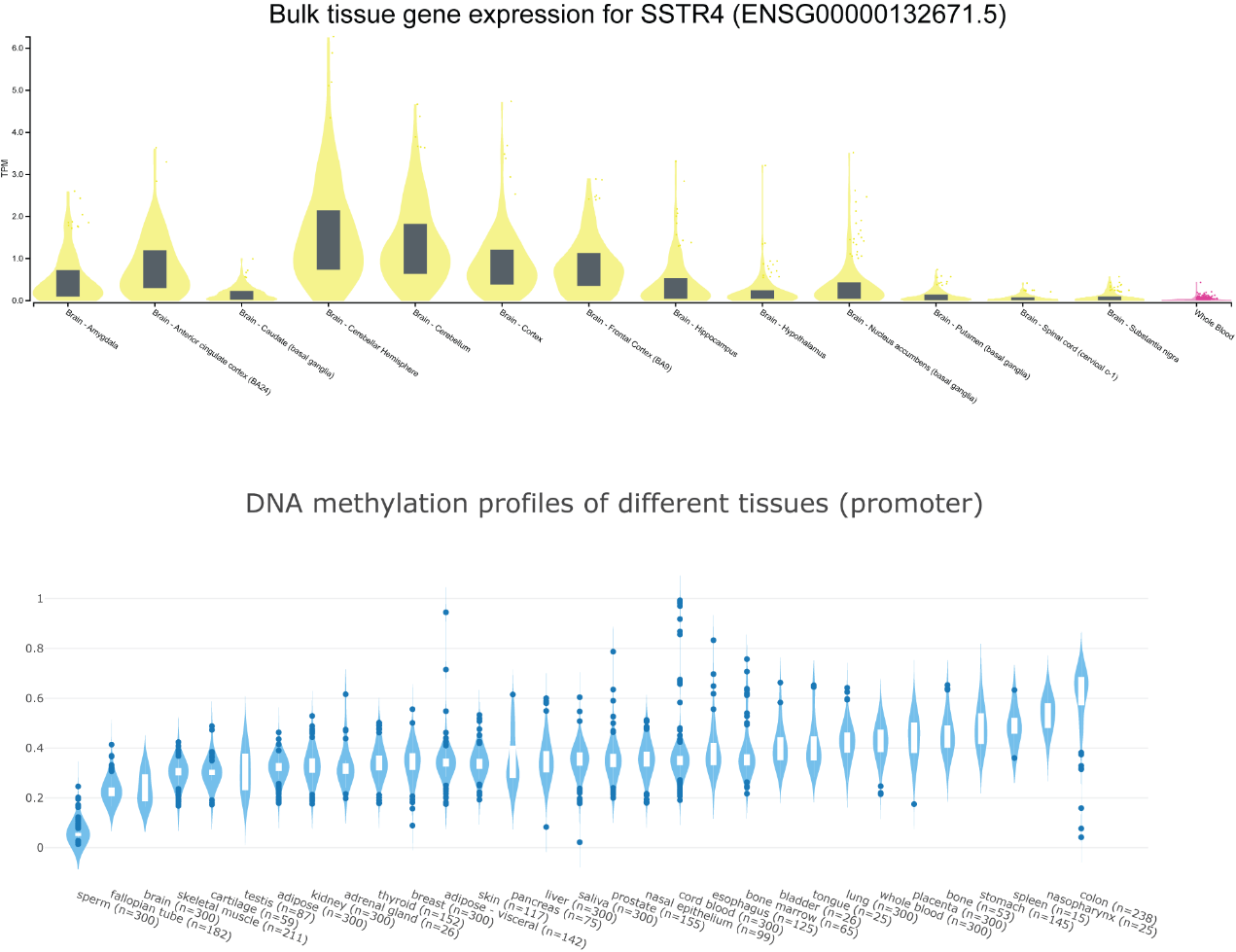


Supplementary Fig S 1

Expression and methylation of *SSTR4* in different tissues.


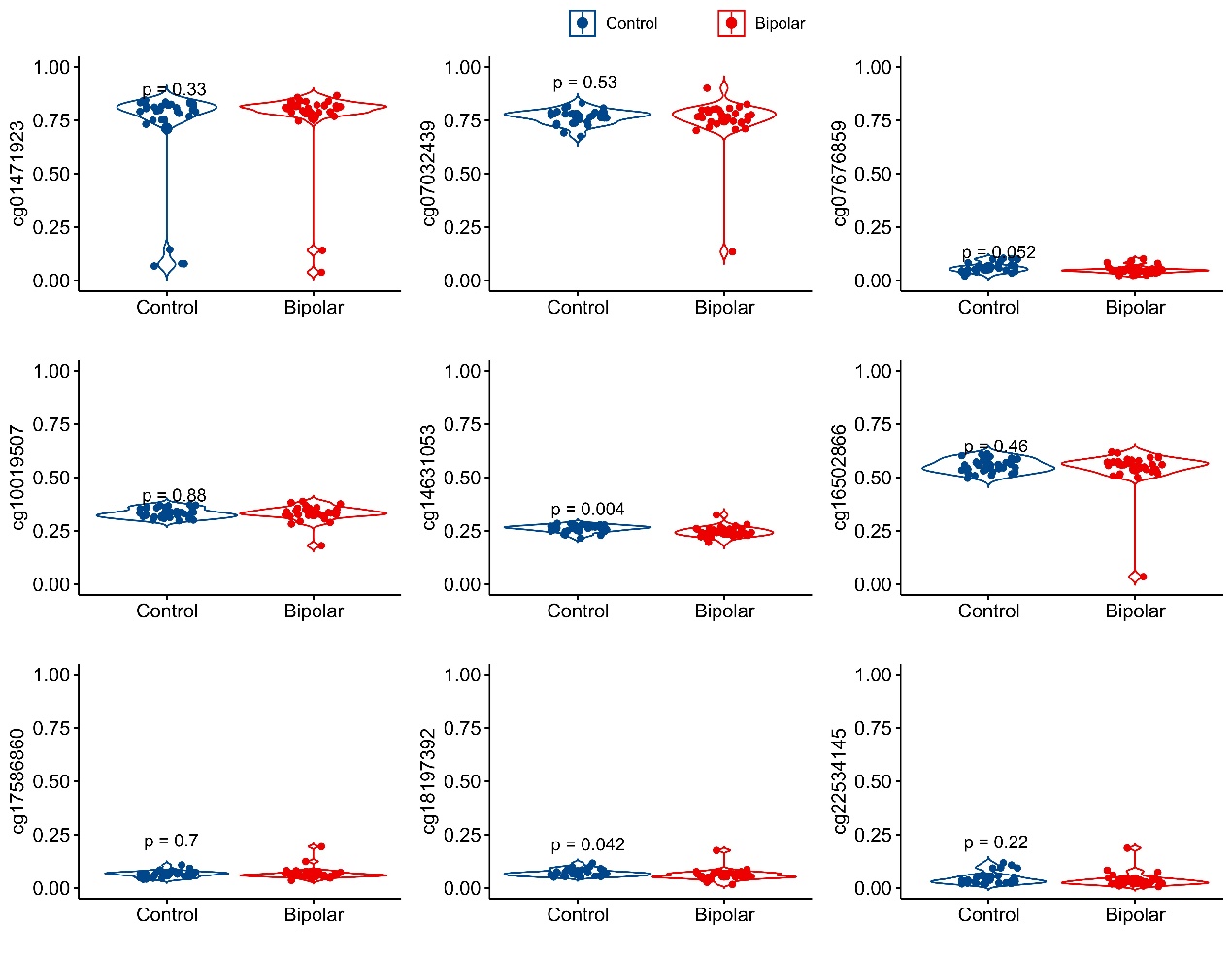


Supplementary Fig S 2

Methylation status comparison of CpG sites from *SSTR4* between healthy control and bipolar


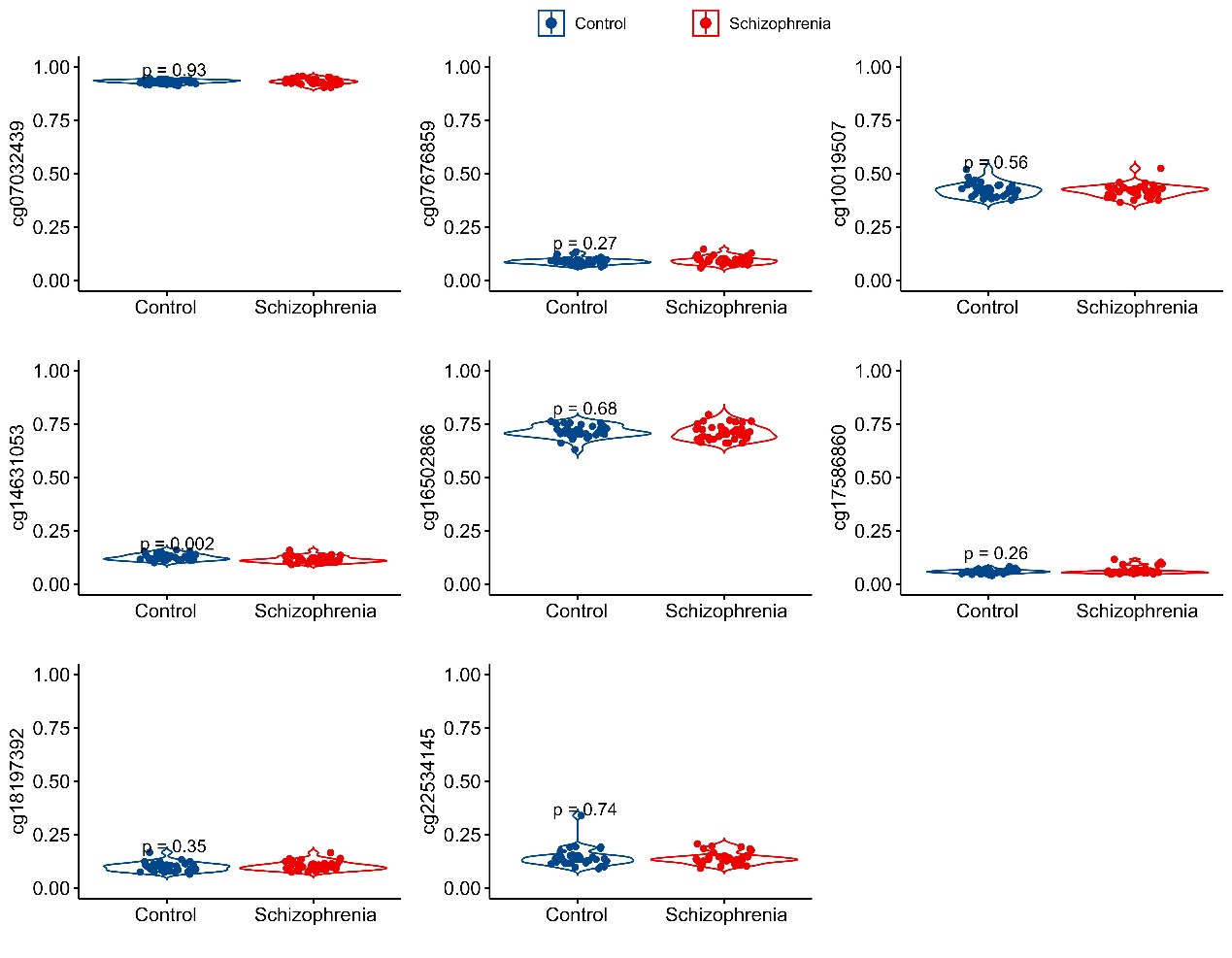


Supplementary Fig S 3Methylation status comparison of CpG sites from *SSTR4* between healthy control and schiophrenia


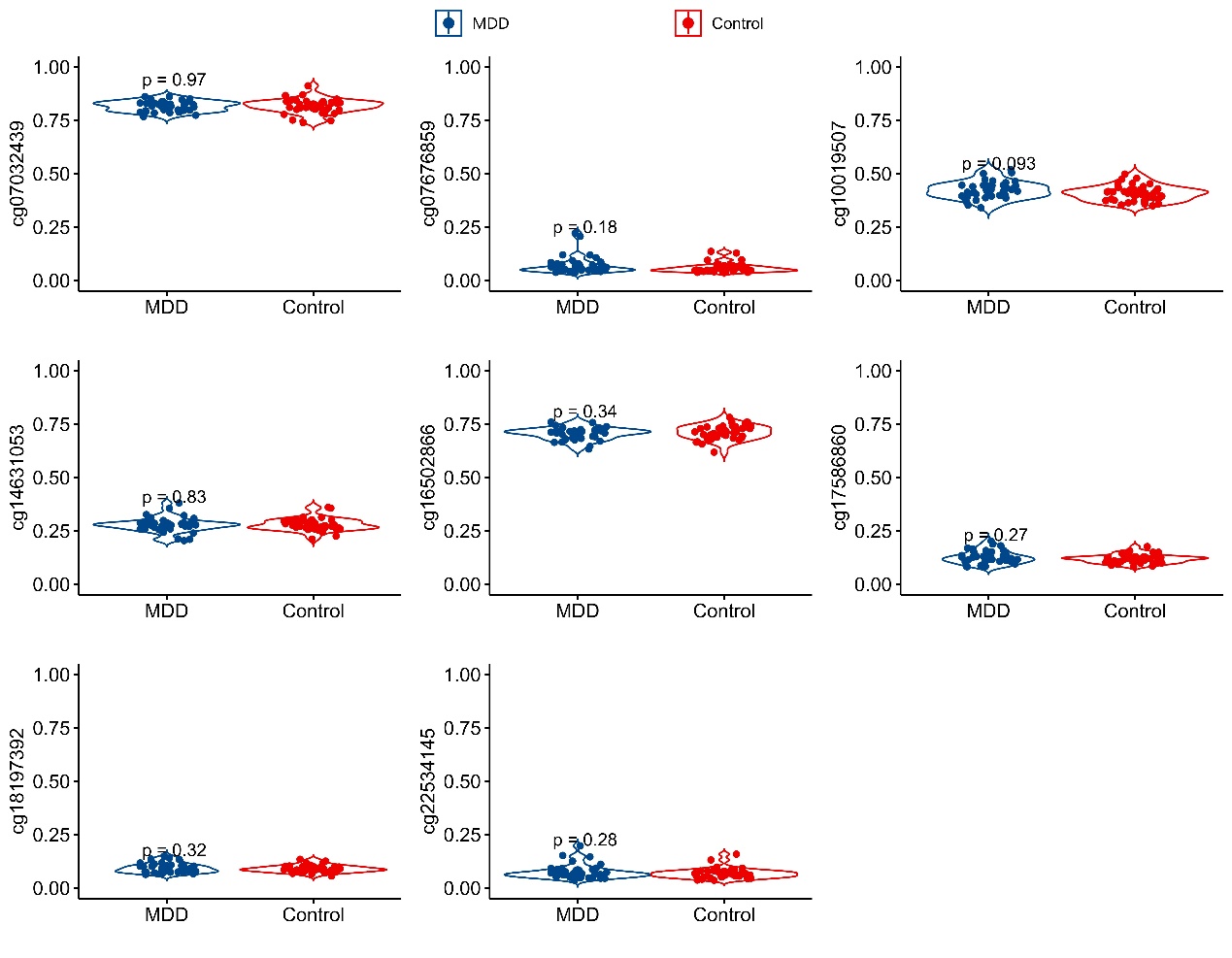


Supplementary Fig S 4Methylation status comparison of CpG sites from *SSTR4* between healthy control and major depressive disorder (MDD)


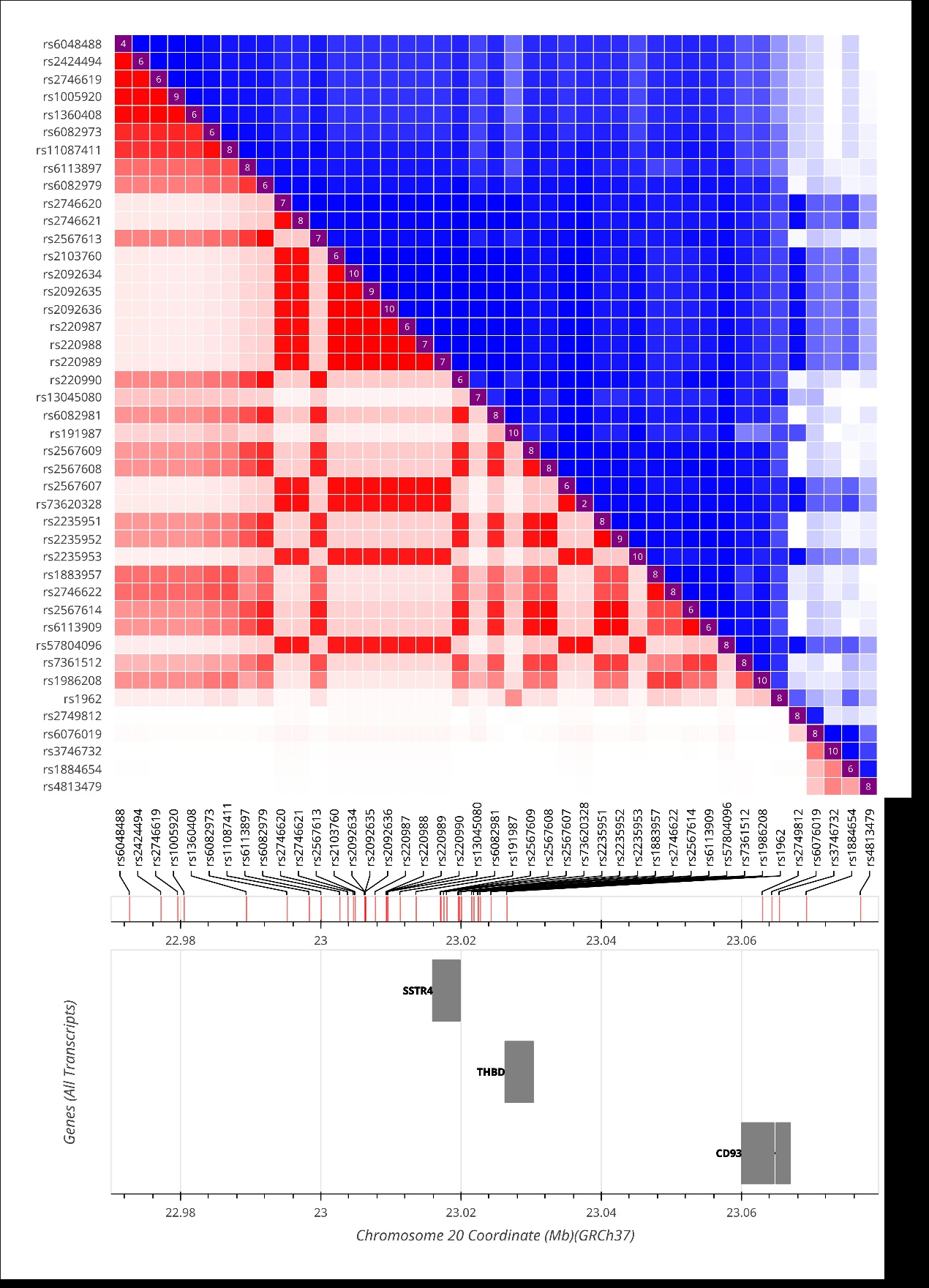


Supplementary Fig S 5Linkage disequilibrium analysis of rs13045080 to other expression quantitative trait loci of *SSTR4*
